# Variations in sexual network connectivity may explain dramatic variations in sexually transmitted infection prevalence between populations and over time: a four-country analysis

**DOI:** 10.12688/f1000research.24968.1

**Published:** 2020-08-20

**Authors:** Chris Kenyon

**Affiliations:** 1 HIV/STI Unit, Institute of Tropical Medicine, Antwerp, Antwerp, 2000, Belgium; 2 Division of Infectious Diseases and HIV Medicine, University of Cape Town, Cape Town, 7700, South Africa

**Keywords:** network connectivity, STI, syphilis, gonorrhoea, herpes simplex virus-2, HIV, chlamydia, epidemiology

## Abstract

**Background:** The incidence of sexually transmitted infections (STIs) has been noted to vary dramatically between population groups and over time. Here, the hypothesis that changes in network connectivity underpin these changes is explored.

**Methods:** The incidence/prevalence estimates of HIV, herpes simplex virus-2, syphilis, chlamydia, and gonorrhoea, as well as two markers of sexual network connectivity (partner concurrency and multiple partnering) by ethnic group and sexual orientation in Kenya, South Africa, the United Kingdom (UK) and the United States (USA) were extracted from published studies. Pearson’s correlation was used to test the association between the markers of network connectivity and the incidence/prevalence of these five STIs. A literature review was performed to evaluate the possible causes of the increases and decreases in syphilis incidence over the past 60 years.

**Results:** In each country, the five STIs were found to cluster in particular ethnic groups and sexual orientations and to be positively associated with the two markers of network connectivity. Syphilis incidence in the UK and USA was found to increase dramatically in the 1960s/1970s, decline in the 1980s and again increase in the late 1990s. These changes took place predominantly in men who have sex with men, and were preceded by corresponding changes in network connectivity. The large decline in antenatal syphilis prevalence in Kenya and South Africa in the 1990s were likewise preceded by declines in network connectivity.

**Conclusions:** Although other explanatory variables are not controlled for, the present analysis is compatible with the hypothesis that differential network connectivity is a parsimonious explanation for variations in STI incidence over time and between populations.

## Introduction

**Figure 1.**
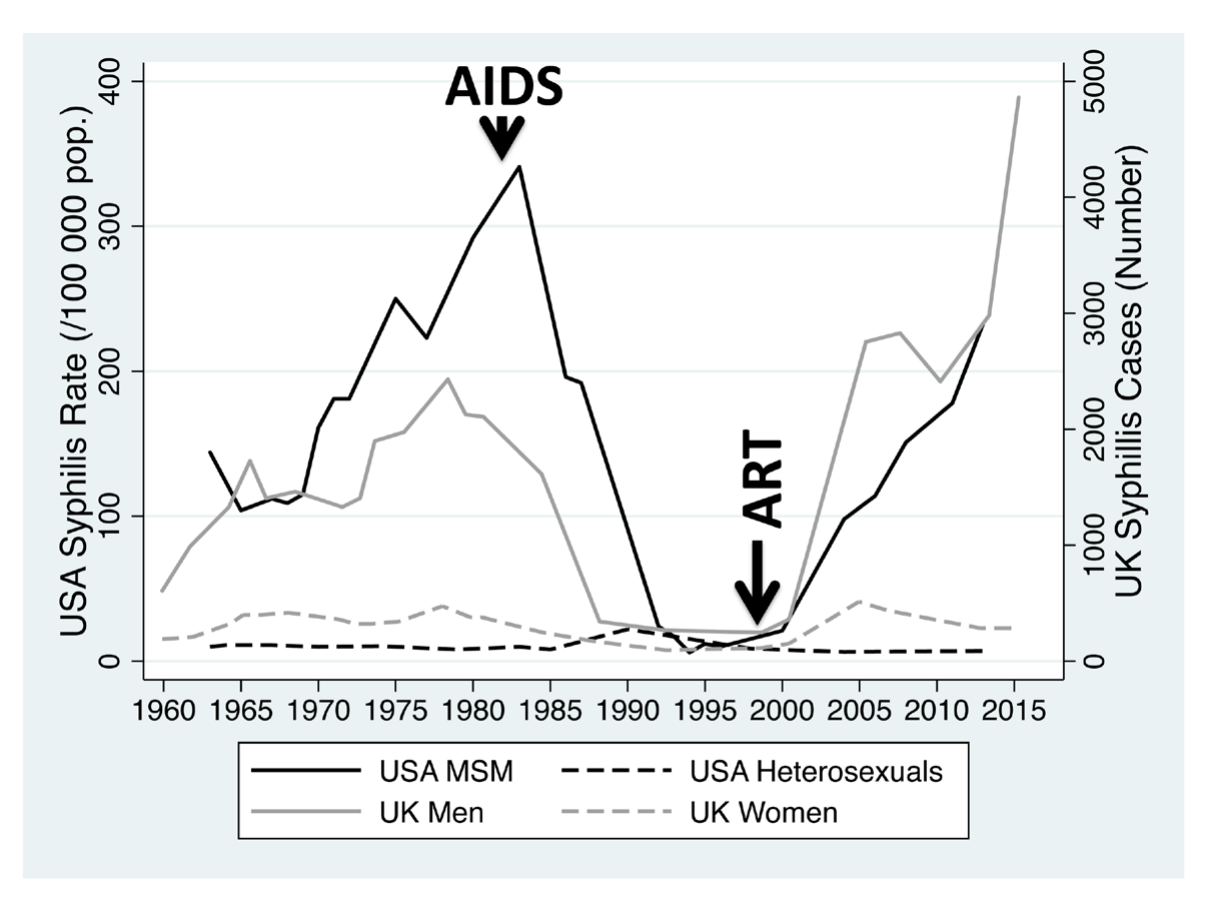
Incidence of primary/secondary syphilis in the United States (MSM and heterosexual men; cases per 100 000/population) between 1963–2013 and cases of primary, secondary and early latent syphilis diagnosed in men and women in England and Wales between 1950 and 2015. Beginning of ‘AIDS’ epidemic and introduction of ‘ART’ – antiretroviral therapy – indicated with arrows.

The reasons underpinning the dramatic variations in sexually transmitted infection (STI) incidence between different populations are incompletely understood ^1, 2^. Not infrequently the incidence of specific STIs varies by an order of magnitude or more between ethnic groups and between groups defined according to sexual orientation ^3– 6^. Furthermore, over the past century, there have also been numerous examples of dramatic changes in the incidence of specific STIs. The incidence of syphilis in men who have sex with men (MSM) in the United States (USA), for example, was high in the 1970s, but declined dramatically in the early 1980s only to return to 1970s levels more recently ( Figure 1) ^7^. In this paper we test the hypothesis that differences in the connectivity of local sexual networks play an important role explaining these cross sectional and longitudinal variations in STI incidence ^8, 9^. Although the connectivity of local sexual networks is determined by a number of parameters, we focus on two of the most important - the rate of partner change and the proportion of partnerships that run concurrently ^8, 9^. Previous analyses have typically found that one, or a combination of markers of network connectivity, are raised in populations with a higher prevalence of a particular STI ^3, 4, 9, 10^. These studies have however typically been limited to single countries and frequently only investigated variations in a single STI and either considered variations between ethnic groups or sexual orientations but not both ^11– 20^. Global country-level analyses have produced a mix bag of results with a small number of studies ^21, 22^ finding positive associations between markers of network connectivity and STI prevalence, and a larger number finding no association ^23– 26^. A possible explanation for this discrepancy is a type of misclassification bias that could be termed a ‘fruit salad bias’ ^22, 27^. It is increasingly appreciated that each country is made up of a number of sexual networks (defined by sexual orientation, ethnicity, class, religion etc.) that interconnect to varying degrees ^2, 4, 9, 28^. If the connectivity of these sexual networks is a major determinant of STI prevalence, then it would be most appropriate to compare the association between network connectivity and STI prevalence between sub-populations of sexual networks within a country rather than combining them all into one national population (fruit salad) as a number of studies have done ^10, 24, 25^.

These insights provided the motivation for the current paper where we aimed to conduct a more systematic analysis of the association between network connectivity and STI prevalence in national subpopulations. We expanded the previously conducted analyses of this association in four ways. Firstly, we assessed this association in four countries: two high income countries (the United Kingdom [UK] and [USA]) where STIs are predominantly concentrated in MSM and certain ethnic groups and two low/middle income countries that have been affected by generalized HIV epidemics (Kenya and South Africa) but where certain ethnic groups have largely escaped this epidemic. Secondly, in each country we assess this association at the levels of both ethnic groups and MSM versus heterosexuals. Thirdly, we assess this association in five STIs (chlamydia, gonorrhoea, HIV, herpes simplex virus-2 [HSV-2] and syphilis). Fourthly, we conduct longitudinal analyses over the past 60 years to assess if increases and decreases in syphilis incidence and prevalence are preceded by corresponding changes in network connectivity.

## Methods

### Data sources

A search was conducted on PubMed and Google Scholar on 15 February 2020 using the following terms: ‘chlamydia’, ‘gonorrhoea’, ‘HIV’, ‘HSV-2’, ‘syphilis’, ‘concurrency’, 'multiple partnering’, ‘Kenya’, ‘South Africa’, ‘UK’, ‘United Kingdom’, ‘USA’, ‘United States’, ‘men who have sex with men’, “MSM”.

***Cross sectional STI data.*** The incidence and prevalence estimates of chlamydia, gonorrhoea, HIV, HSV-2, syphilis, concurrency and multiple partnering in Kenya, South Africa, the UK, and USA were extracted from published studies, as detailed in Table 1 and Table 2. When two or more studies were found, we favoured data from studies based on nationally representative samples. In addition, for the cross-sectional analyses, we limited studies to those based on data from after the year 1990 and favoured the most recent data.

**Table 1.** Description of sources of data for comparison between ethnic groups: prevalence of STIs, multiple partners, concurrency.

Country	Year data collected, reference	Study type, selection procedure, sample size
**Kenya**		
HIV, HSV-2 & syphilis	2007 ^29^	The 2007 Kenya AIDS Indicator Survey used a stratified two stage sampling strategy to test a nationally and provincially representative sample of 15 853 15 to 64 year olds.
Male urethral discharge & circumcision	2008 ^30^	The 2008 Kenya Demographic and Health Survey used a household-based, two- stage stratified sampling approach to recruit 12 677 participants.
Concurrency & multiple partners	2011 ^31, 32^	The Population Services International (PSI) Survey /Kenya 6th HIV Survey conducted in 2011 used a two stage cluster sampling to obtain a provincially representative sample of households from seven of Kenya’s eight provinces (the North East was excluded). A total of 3 051 men and women 15-49 year old were included.
**South Africa**		
HIV	2005 ^33^	A two-stage, nationally representative sample of 23 275 persons 2 years old or older. We limited our analysis to the 13 884 individuals aged 15 to 49 years old.
HSV-2	2012 ^34^	The prevalence of HSV-2 was assessed in the 2012 national antenatal HIV and HSV-2 survey. The prevalence of HSV-2 was estimated for 18 732 women in four provinces - Gauteng, KwaZulu-Natal, Northern Cape and Western Cape. The Focus HerpesSelect 2 ® ELISA IgG Diagnostic kit was used as the diagnostic test.
Syphilis	1991 ^35^	A sample of 17 318, 15-49 year old women attending antenatal clinics for their first visit were tested for syphilis via the RPR or VDRL test.
Male urethral discharge	1998 ^36^	The 1998 Demographic and Health Survey employed a 2-stage sampling strategy in South Africa’s nine provinces and stratified results into urban and non-urban groups. It was designed to be representative for all provinces and the four major ethnic/racial groups. 6578 men were asked if they had experienced symptoms of a urethral discharge in the last 3 months. Men were not asked questions about their sexual behaviour in this survey.
Concurrency, Multiple partners & circumcision	2003 ^4, 37^	The 2003 Demographic Health Survey (DHS) used a similar study design to the 1998 DHS. The survey sampled 7966 women and 3930 men. All were 15–49 year old.
**United Kingdom**		
HIV & Male urethral discharge	2010–2012 ^19^	National Surveys of Sexual Attitudes and Lifestyles 3 recruited a probability sample of 15 162 women and men aged 16–74 years in Britain. Participants were interviewed with computer-assisted face-to-face and self-completion questionnaires. Urine from a sample of participants aged 16–44 years who reported at least one sexual partner over the lifetime was tested for HIV antibodies.
Chlamydia, gonorrhoea & syphilis	2018 ^38^	The incidence figures for these three STIs by ethnic group were extracted from the Public Health England report for 2018: Sexually transmitted infections and screening for chlamydia in England. The figures were reported separately for men and women but not combined. We report the results for men. The rank order of each STI incidence by ethnic group did not differ by sex ^38^. The results are reported as infections per 100 000 population per year.
Concurrency, multiple partners, STI symptoms	2000 ^39, 40^	The second British National Survey of Sexual Attitudes and Lifestyles (NATSAL 2) was a nationally representative sample of 11161 men and women aged 16-44 years ^39^. We extracted the relevant variables from a study which broke down the various sexual behaviour variables by ethnic group and sex ^39^. Men were asked if they had ever been diagnosed with an STI.
**USA**		
HIV	2006 ^41^	HIV prevalence at the end of 2006 was estimated using information from the national HIV/AIDS Reporting System.
HSV-2	2005-2008 ^42^	HSV-2 seroprevalence was assessed in a nationally representative sample of 7 293 persons 14-49 years in the NHANES 2005-2008 sample.
Syphilis	2001-2004 ^43^	Sera from 5767, 18- to 49-year-old participants in the NHANES 2001–2004 were tested for syphilis IgG antibody using an enzyme immunoassay (EIA). Specimens with positive or indeterminate EIAs underwent rapid plasma reagin (RPR) testing.
Gonorrhoea & Chlamydia & Syphilis	2017 ^44^	The incidence estimates for gonorrhoea, chlamydia and syphilis are based on the CDCs 2017 STD Surveillance Report. They are reported as infections per 100 000 population per year.
Concurrency & Multiple partners	1992 ^28^	The prevalence of concurrency and multiple partners were taken from the 1992 National Health and Social Life Survey (NHSLS). This was a cross-sectional study that used a nationally representative stratified random sample of 3 432 women and men between the ages of 18 and 59.

Abbreviations: STI – sexually transmitted disease, DHS - Demographic and Health Survey, NATSAL - National Survey of Sexual Attitudes and Lifestyles, NHANES - National Health and Nutrition Examination Surveys.

**Table 2.** Description of sources of data for comparison between men who have sex with men and men who have sex with women: prevalence of STIs, multiple partners and concurrency.

Country	Year data collected, reference	Data source. Study type, selection procedure, sample size
**USA**		
HIV	2009-12 ^52^	HIV prevalence estimates for MSM and hetero sexual men were taken from an analysis of the National Health and Nutrition Examination Survey (NHANES) 2009–2012 survey. Men who reported ever having had sex with another man were classified as men who have sex with men (MSM). Men who reported no sex with other men were classified as heterosexual men.
Chlamydia, gonorrhoea and syphilis	2013 ^7, 44^	CDC STI surveillance reports note that with the exception of reported syphilis cases, nationally notifiable STI surveillance data do not routinely include information on sexual partners/orientation., As a result this data is missing for the majority of gonorrhoea and chlamydia cases reported to CDC ^44^. No estimates are therefore provided for chlamydia or gonorrhoea incidence. The primary and secondary syphilis incidence estimates for 2013 (reported as cases per 100 000 population per year) are taken from a paper that estimated incidence in MSM and heterosexual men based on cases of primary and secondary syphilis reported to the CDC between 1963 and 2013 7.
Concurrency, multiple partners	1996-2006 ^50^	Sexual behaviour data was taken from a paper that compared sexual behaviour patterns between MSM and male and female heterosexuals aged 18–39 in the USA. Their data was obtained from 4 population-based random digit dialling surveys. A 1996–1998 survey in 4 U.S. cities and 2 Seattle surveys (2003, 2006) provided estimates for MSM; a 2003–2004 Seattle survey provided data about heterosexual men and women. The multiple partner variable refers to the mean lifetime number of partners reported by respondents (aged 35-39 years). Concurrency was defined as the respondent reporting two or more sexual partnerships with overlapping dates in the prior year ^50^.
**United Kingdom**		
HIV	2016 ^53^	HIV prevalence estimates were taken from the Public Health England’s report ‘HIV in the UK, 2016’ 53. These figures are based on a Bayesian multi-parameter evidence synthesis model, which combines data from multiple sources to provide prevalence estimates for HIV for different sub populations ^53^.
Chlamydia, gonorrhoea and syphilis	2018 ^38^	The incidence figures for these three STIs were taken from Public Health England’s report ‘Sexually transmitted infections and screening for chlamydia in England, 2018’ ^38^. This report provides incidence estimates for chlamydia, gonorrhoea and syphilis for heterosexual men, heterosexual women, HIV-infected MSM and ‘HIV-uninfected or HIV-undiagnosed MSM’ in 2018. We report the results for the first and last two of these categories. There was little difference in the incidence estimates for heterosexual men and women ^38^.
Concurrency, multiple partners	2012 ^51^	The third British National Survey of Sexual Attitudes and Lifestyles (NATSAL 3) was a nationally representative sample of 15,162 men and women aged 16-74 years conducted in 2012 ^51^. We extracted the relevant variables from a study report which provided prevalence estimates of sexual behaviour for MSM and heterosexual men. The multiple partnering variable referred to the mean lifetime number of sexual partners reported by respondents. The proportion reporting overlapping sexual partnerships at any point in the past 5 years was used as the measure of concurrency.

**Table 3.** Description of sources of data for longitudinal changes in syphilis incidence and prevalence.

	Year data collected, reference	Data source. Study type, selection procedure
**Kenya**	1992-1997 ^68^	81 311 pregnant women from 10 antenatal sentinel sites in Nairobi were screened for syphilis on their first antenatal visit using the RPR only. Results were used to generate annual syphilis prevalence estimates for each year 1992 to 1997. ^68^
**South** **Africa**	1938 to 2011 ^45^	Longitudinal syphilis prevalence estimates were taken from a global epidemiology of syphilis over the past century paper. Seventeen studies, all performed in antenatal populations, evaluated the prevalence of syphilis in pregnant women between 1938 and 2011 ^45^
**UK**	1960 to 2015 ^1^	The longitudinal syphilis incidence estimates are for the annual number of new cases of primary, secondary and early latent syphilis diagnosed in men and women in England and Wales between 1960-2015. The data is also based on case reporting surveillance data and is from the Lancet Infectious Diseases Commission: Sexually transmitted infections: challenges ahead ^1^.
**USA**	1963 to 2013 ^7^	The primary and secondary syphilis incidence estimates for 1963 to 2013 (reported as cases per 100 000 population) are taken from a paper that estimated incidence in MSM and heterosexual men based on cases of primary and secondary syphilis reported to the CDC in this time period ^7^.

*Symptomatic STI:* In the case of Kenya and South Africa, we could not find high quality data on STI prevalence, or behaviour reported by sexual orientation. In addition, we could not find reasonable quality data reporting the incidence/prevalence of chlamydia or gonorrhoea by ethnic group in these two countries. As a result, we use the proportion of men reporting urethral discharge as a composite proxy for combined chlamydia plus gonorrhoea incidence per ethnic group ^30, 36^. 

***Longitudinal syphilis data.*** We chose syphilis for the evaluation of longitudinal changes in STI prevalence for a number of reasons, including the fact that syphilis surveillance programmes from the four countries have provided reasonable quality estimates of syphilis incidence for the last 60 years and longer – a far longer period than that for which reasonable incidence estimates are available for other STIs ^45^. To a large extent, this is related to the fact that relatively accurate serological tests have been available for the diagnosis of syphilis for longer than other STIs and these have been extensively used to diagnose antenatal syphilis ^45– 47^. Antenatal syphilis surveys have been shown to produce reasonable estimates of the prevalence of syphilis in the general population ^45– 47^. For Kenya and South Africa, we used large antenatal syphilis prevalence surveys - national in the case of South Africa and limited to the capital in the case of Kenya ( Table 1). For the UK and USA, we used incidence estimates based on national case reporting data ( Table 1). In the UK, the sexual orientation of individuals with syphilis was not available for much of this period and thus we follow the example of others who compare the ratio of the incidence in men to that in women as a proxy for the incidence in MSM ^1, 48, 49^.

***Multiple partners and concurrency.*** Studies use a wide variety of definitions of multiple partnering and concurrency.

*Concurrency:* In all the comparisons of ethnic groups, excluding the UK, the prevalence of concurrency was defined as the percentage of men (15–49 years old) who reported having two or more sex partners at the time of the survey. In the UK, concurrency referred to the percentage of men who reported any overlapping sexual relationships in the past year ^39^. In the comparisons of sexual orientations, for the USA, concurrency was defined as the respondent reporting two or more sexual partnerships with overlapping dates in the prior year ^50^. As far as the UK was concerned, this referred to the proportion reporting overlapping sexual partnerships at any point in the past 5 years ^51^.

*Multiple partners:* For the ethnic group comparisons, the prevalence of multiple partners was defined as the percentage of men (15–49 years old) who reported having two or more sex partners in the last year. In the UK, this variable was not reported by ethnic group. As a result, multiple partnering here refers to the percentage of men who reported one or more new heterosexual relationship in the prior year. For the MSM vs. heterosexual comparisons, this variable referred to the mean lifetime number of sexual partners reported by respondents in the UK (aged 16–74) and the USA (aged 35–39).

These variations in definitions between studies mean it would be appropriate to compare these variables between studies. Since all the studies we used were limited to specific countries, we only compare subpopulations within each study/country.

***Regions vs. ethnic group in Kenya.*** Many of the national STI surveillance surveys in Kenya do not collect data on ethnic group ^29^. There is however a high correlation between region of residence and ethnic group, with large differences in STI incidence and sexual behaviour between regions ^5, 31^. We therefore follow the approach used in previous studies and use region of residence as a proxy for ethnic group ^31, 54^.

### Statistical analysis

We report the incidence/prevalence of the five STIs, multiple partnering and concurrency by ethnic group (all four countries) and MSM vs. heterosexuals in each country with available data (UK and USA). Where this data is provided, we report 95% confidence intervals. We used the Chi-squared test to assess if there was a difference in incidence/prevalence between the subgroups with the highest and lowest incidence/prevalence values in each country. For continuous variables (lifetime number of partners) the Man-Whitney U test was used. The correlation between the prevalence of the different STIs per subgroup in each country, and between the prevalence of each STI and the two behavioural variables was calculated using Pearson’s correlation. Because of the small sample sizes (between 2 and 8), tests of statistical significance were not performed for these calculations. In the case of UK and USA, the STI data is presented as an estimated incidence per 100 000 per year without reporting the number of cases and the total size of the study populations. As a result, we did not test if the association between the prevalence of each STI and each of the markers of network connectivity is statistically significant. The key differences were however large, which allows substantive inferences to be drawn. Where data was only provided in graphical form, we digitized the data using GetData Graph Digitizer version 2.26. STATA v15.2 was used for all analyses (StataCorp LLC, College Station, TX, USA).

### Longitudinal association between network connectivity and syphilis prevalence/incidence

A literature review was performed to evaluate the possible causes of increases and decreases in syphilis incidence in each subpopulation. We used a narrative approach here to evaluate if there was evidence from the literature that increases and decreases in syphilis prevalence were preceded by corresponding changes in markers of network connectivity. The approach adopted was not to perform a systematic review or a quantitative analysis, but rather to assess if there is evidence in the literature that corroborates or refutes the network connectivity hypothesis.

## Results

### Cross sectional analyses

***Kenya***

**Ethnic group**

**Figure 2.**
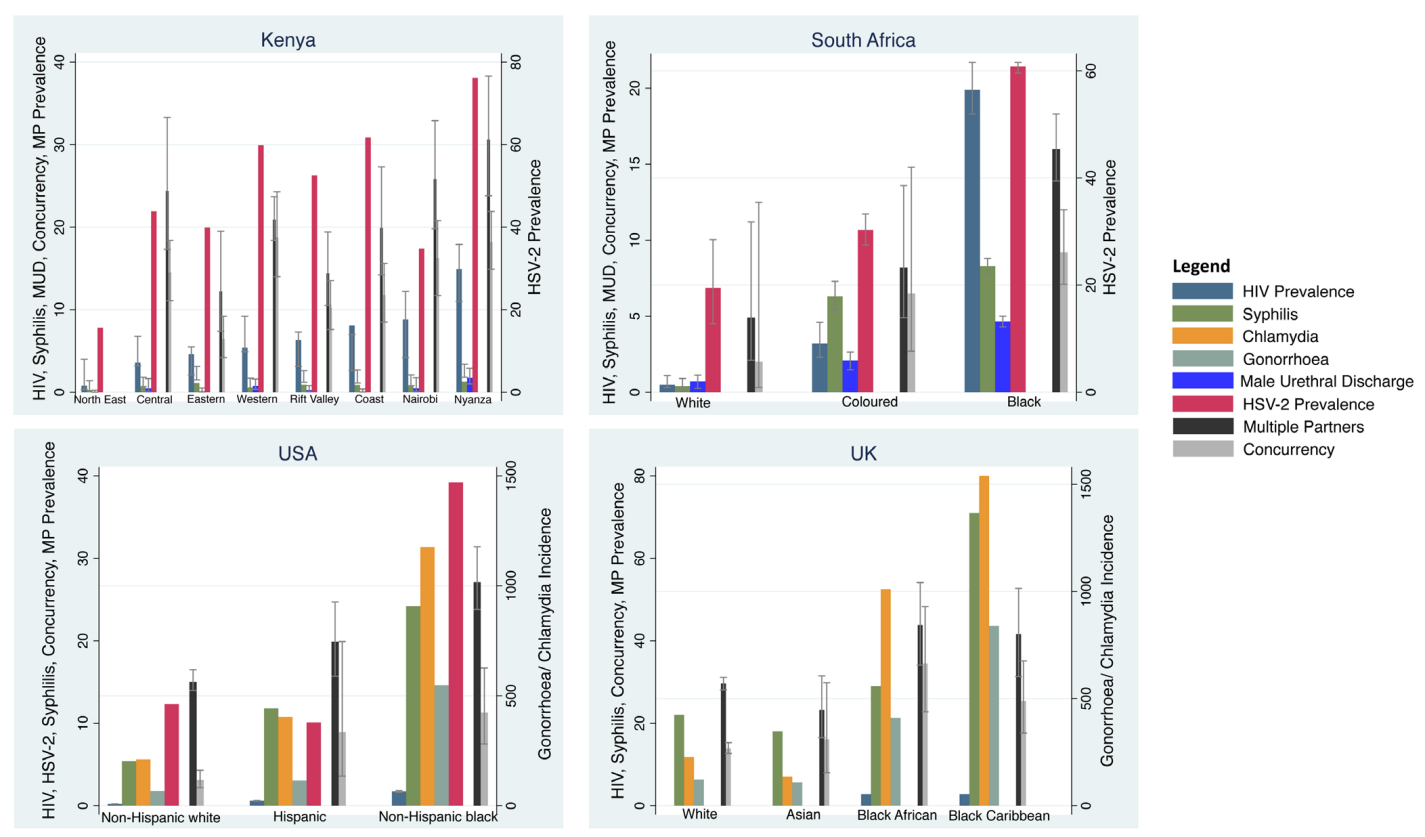
Incidence/prevalence of HIV, HSV-2, chlamydia, gonorrhoea, syphilis, male urethral discharge (MUD), partner concurrency and multiple partnering (MP) by ethnic group in Kenya, South Africa, the United Kingdom and the United States of America. For all countries, HIV, HSV-2, male urethral discharge, multiple partners and concurrency prevalence are reported as percentages. The same is true for syphilis in Kenya and South Africa. In the USA and the UK, chlamydia, gonorrhoea and syphilis are reported as cases per 100 000 per year (Point estimates with 95% Confidence Intervals; see Table 1 for sources of data).

HIV prevalence varied 15-fold between 0.9% (95% confidence interval [CI] 0.3-4%) in the North East and 13.9% (95% CI 11.0-17.9%) in Nyanza ( Figure 2; Table 1, Table 4 & Table 7). HSV-2 prevalence varied 5-fold between 15.6% and 76.2% in these same two regions. The prevalence of syphilis varied 5-fold between 0.5% (95% CI 0.2-1.4%) and 2.5% (95% CI 1.8-3.4%) in these same two regions. Likewise, the percentage of men reporting urethral discharge in the prior year varied between 0.0% (95% CI 0.0-0.5%) and 3.5% (95% CI 2.0-5.8%; all P<0.001) in the same two regions. The prevalence/incidence of male urethral discharge, HIV, HSV-2 and syphilis by region were positively correlated with each other (r-0.35 to r-0.77; Figure 2, Table 6).

**Table 4.** Prevalence/incidence of HIV, HSV-2, chlamydia, gonorrhoea, syphilis, concurrency and multiple partnering by ethnic group in Kenya, South Africa, the United Kingdom and the Unites States.

Country	Ethnic group	HIV (%)	HSV-2 (%)	Chlamydia ^$^	Gonorrhoea ^$^	Syphilis ^+^	STI symptoms ^#^	Concurrency (%) ^#^	Multiple partners (%) ^#^
**SA**	White	0.5 (0.3-1.1)	6.3	NA	NA	0.4 (0.1-0.9)	2.0 (0.7-3.2)	2.0 (0.3-12.5)	4.9 (2.1-11.2)
	Coloured	3.2 (2.3-4.6)	16.7	NA	NA	6.3 (5.4-7.3)	5.9 (4.2-7.5)	6.5 (2.7-14.8)	8.2 (4.9-13.6)
	Black	19.9 (18.3-21.7)	31.3	NA	NA	8.3 (7.8-8.8)	13.2 (12.2-14.2)	9.2 (7.1-12.0)	16 (13.9-18.3)
**Kenya**	Nairobi	7.2 (4.2-12.2)	34.8	NA	NA	1.4 (0.8-2.1)	0.91 (0.23-3.5)	16.0 (11.7-20.8)	25.8 (19.8-32.9)
	Central	4.6 (3.2-6.8)	43.8	NA	NA	1.2 (0.7-1.8)	0.88 (0.22-3.3)	14.5 (11.1-18.4)	24.4 (17.3-33.3)
	Coast	4.2 (2.6-7.0)	61.8	NA	NA	1.8 (1.1-2.7)	0.18 (0.04-0.77)	11.7 (8.5-15.6)	19.9 (14.2-27.3)
	Eastern	3.5 (2.1-5.5)	39.9	NA	NA	2.2 (1.5-3.1)	0.24 (0.05-1.0)	6.4 (4.2-9.2)	12.2 (7.4-19.5)
	Nyanza	13.9 (11.0-17.9)	76.2	NA	NA	2.5 (1.8-3.4)	3.45 (2.0-5.8)	18.2 (14.9-21.9)	30.6 (23.8-38.3)
	Rift Valley	4.7 (3.1-7.3)	52.6	NA	NA	1.8 (1.2-2.6)	0.54 (0.2-1.6)	10.3 (7.6-13.5)	14.4 (10.5-19.4)
	Western	6.6 (4.9-9.2)	59.9	NA	NA	1.0 (0.5-1.7)	1.52 (0.7-3.2)	18.8 (14.0-24.3)	20.9 (18.4-23.7)
	North Eastern	0.9 (0.3-4.0)	15.6	NA	NA	0.5 (0.2-1.4)	0 (0-0)	NA	NA
**UK**	White	0	NA	227	121	22	NA	13.9 (12.7-15.3)	29.6 (28.1-31.1)
	Black Caribbean	2.8	NA	1540	840	71	NA	25.4 (17.6-35.1)	41.6 (31.3-52.7)
	Black African	2.8	NA	1011	410	29	NA	34.5 (22.8-48.3)	43.8 (34.1-54.1)
	Asian	0	NA	136	109	18	NA	16.1 (8.0-29.8)	23.2 (16.5-31.5)
**USA**	Non- Hispanic white	0.22 (0.21-0.24)	17.6	211	66	5.4	NA	3.1 (2.2-4.3)	15.0 (14.0-16.5)
	Hispanic	0.59 (0.53-0.64)	22.3	404	114	11.8	NA	8.9 (3.6-19.9)	19.9 (15.7-24.7)
	Non- Hispanic black	1.72 (1.61-1.82)	45.9	1176	548	24.2	NA	11.3 (7.5-16.7)	27.1 (23.8-31.4)

NA – Not Available ^$ ^Incidence in cases per 100 000 population per year ^+^ Incidence for Kenya is percent positive of 15–49 year old population, for South Africa is percent antenatal population positive and for UK and USA is cases per 100 000 population per year ^# ^The definitions used for STI, symptoms, concurrency and multiple partners varied between studies – see Table 1 for details

The region with the lowest HIV prevalence (the North East) was not included in the behavioural survey that provided our data. Other data sources have however found the rate of multiple partnering in this region to be very low ^5^. In our data, the region with the second lowest HIV prevalence (Eastern) had the lowest reported prevalence of multiple partnering (12.2%; 95% CI 7.4-19.5%) and concurrency (6.4%; 95% CI 4.2-9.2%; all P<0.001). Nyanza reported the highest prevalence of multiple partnering (30.6%; 95% CI 23.8-38.3%) and the second highest prevalence of concurrency (18.2%; 95% CI 14.9-21.9%).

With the exception of syphilis, which was negatively associated with concurrency prevalence (r- -0.34), the prevalence of each STI was positively correlated with both the multiple partnering (r-0.38 to r-0.84) and concurrency (r-0.42 to r-0.72) variables.

***South Africa***

*Ethnic group*

HIV prevalence varied 40-fold between 0.5% (95% CI 0.3-1.1%) in the White group and 19.9% (95% CI 18.3-21.7%) in the Black group ( Figure 1; Table 4). HSV-2 prevalence varied 5-fold from 6.3% to 31.3% between the same groups. The prevalence of syphilis varied 21-fold between 0.4% (95% CI 0.1-0.9%) and 8.3% (95% CI 7.8-8.8%) in these same two groups. Likewise, the percentage of men reporting urethral discharge in the prior year varied 7-fold between 2.0% (95% CI 0.7-3.2%) and 13.2% (95% CI 12.2-14.2%) in the same groups (all P<0.001). The prevalence/incidence of male urethral discharge, HIV, HSV-2 and syphilis by ethnic group were positively correlated with each other (r-0.78 to r-0.98; Figure 2, Table 6).

The Black group reported a 5-fold higher prevalence of concurrency and a 3-fold higher prevalence of multiple partners than the White group (9.2% ,95% CI 7.1-12.0% vs. 2.0%, 95% CI 0.3-12.5% and 16.0%, 95% CI 13.9-18.3% vs. 4.9% ,95% CI 2.1-11.2%, respectively; all P<0.001). The prevalence of each STI was positively correlated with both the multiple partnering (r-0.87 to r-1.00) and concurrency (r-0.85 to r-0.99) variables ( Table 6).

***USA***

**Ethnic group** HIV prevalence varied 8-fold between 0.2% (95% CI 0.2-0.2%) in the non-Hispanic White group and 1.7% (95% CI 1.6-1.8%) in the non-Hispanic Black group ( Figure 2, Table 4). The incidence of syphilis varied 4-fold between 5 and 24 per 100 000 per year in these same two groups. Likewise, the incidence of chlamydia varied 5-fold and that of gonorrhoea 8-fold between the same groups (all P<0.001). The prevalence/incidence of chlamydia, gonorrhoea, HIV, HSV-2 and syphilis by ethnic group were strongly positively correlated with each other (r-0.97 to r-1.00; Table 6).

The non-Hispanic Black group reported 4-fold higher prevalence of concurrency and a 2-fold higher prevalence of multiple partners than the non-Hispanic Whites group (11.3%, 95% CI 7.5-16.7% vs. 3.1%, 95% CI 2.2-4.3% and 27.1%, 95% CI 23.8-31.4% vs. 15.0%, 95% CI 14.0-16.5%, respectively; all P<0.001). The prevalence of each STI was positively correlated with both the multiple partnering (r-0.94 to r-0.98) and concurrency (r-0.79 to r-0.91) variables.

**Figure 3.**
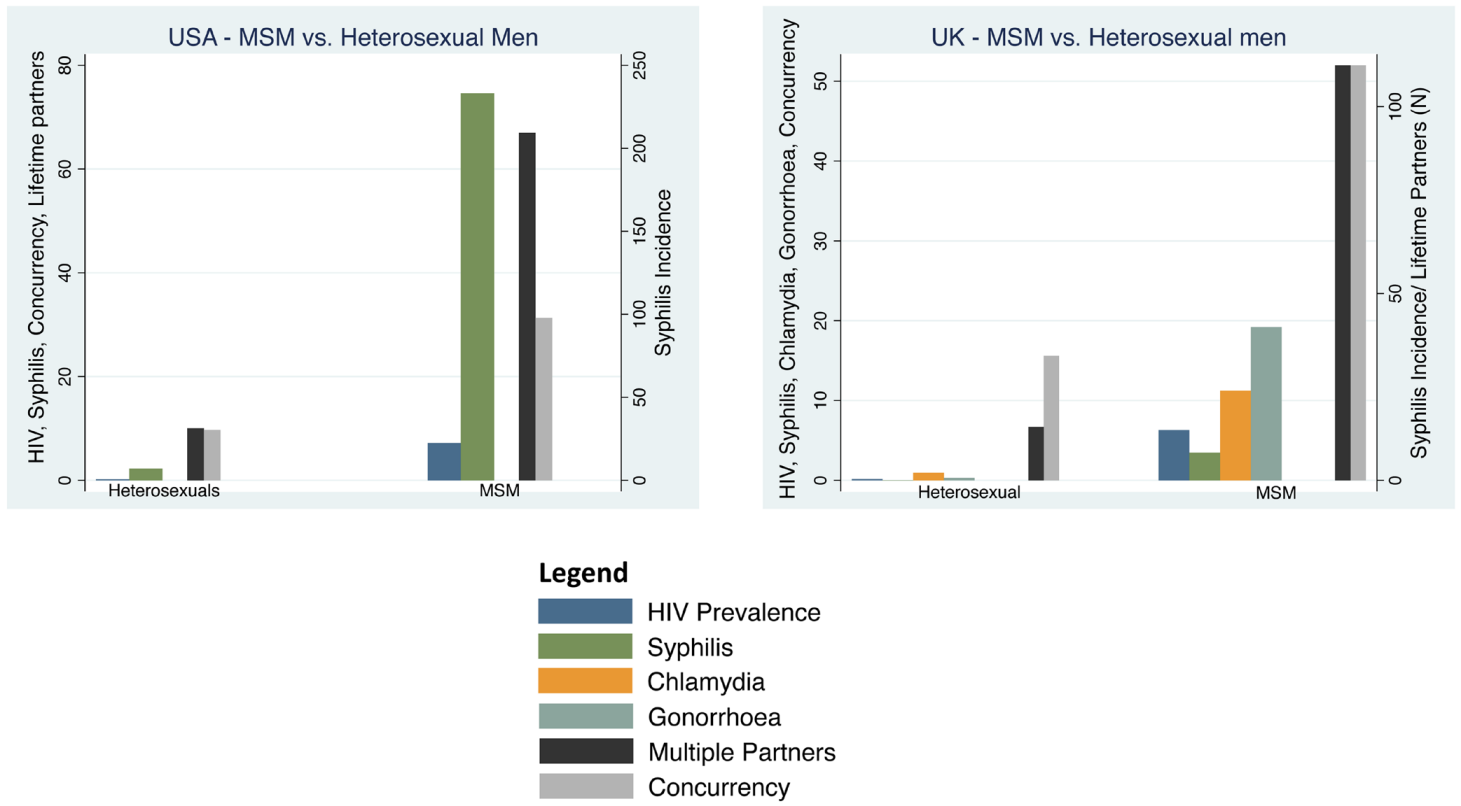
Incidence/prevalence of HIV, chlamydia, gonorrhoea, syphilis, partner concurrency and multiple partnering by men who have sex with men (MSM) versus heterosexual men in the United Kingdom and the United States of America. HIV and partner concurrency are reported as percentages, multiple partners as the number of lifetime partners and chlamydia gonorrhoea and syphilis as cases per 100 000 per year (Point estimates with 95% Confidence Intervals; see Table 1 for sources of data).

**MSM vs heterosexuals** The prevalence of HIV was 36-fold higher (7.2% vs. 0.2%) and the incidence of syphilis 33-fold higher (233 vs. 7/100 000 per year) in MSM compared to heterosexuals ( Table 2 & Table 5, Figure 3). The percent reporting concurrency was 3 times higher in MSM than heterosexuals (31.3% vs. 9.7%). Likewise, the mean number of lifetime partners was 7-times higher (67 vs. 10) in MSM.

**Table 5.** Prevalence/incidence of HIV, HSV-2, chlamydia, gonorrhoea, syphilis, concurrency and multiple partnering by men who have sex with men (MSM) versus heterosexual men the United Kingdom and the Unites States.

Country	Group	HIV %	Chlamydia ^$^	Gonorrhoea ^$^	Syphilis ^$^	Concurrency % (95% CI)	Multiple partners	Lifetime partners ^+^
**USA**	MSM	7.2	NA	NA	233	31.3	86%	67
	Heterosexual men	0.2	NA	NA	7	9.7	56%	10
**UK**	MSM	6.3 (13.4) ^#^	24 (80) ^#^	41 (108) ^#^	7.4 (42.4) ^#^	52.4 (43.5-61.1)	24	111.1
	Heterosexual men	0.16	2	0.6	0.04	15.6 (14.5-16.7)	3.8	14.3

NA- Not Available ^$^ Incidence in cases per 100 000 population per year. ^#^ The incidence estimates are provided for MSM living outside of London and in parentheses the incidence figures for MSM living in London. ^+^ The mean number of lifetime partners reported by 35–39 years olds in the USA and aged 16–74 in the UK.

**Table 6.** Associations between incidence/prevalence of HIV, HSV-2, syphilis, gonorrhoea, chlamydia, male urethral discharge, concurrency and multiple partnering by ethnic group in (A) Kenya and South Africa; and (B) the United Kingdom and United States (Pearson’s Correlations).

Section A							
	HIV	HSV-2	Syphilis	Urethral Discharge	Concurrency	Multiple Partners	
**Kenya**							
HIV	1.00						
HSV-2	0.65	1.00					
Syphilis	0.63	0.39	1.00				
Uretdral Discharge	0.77	0.68	0.34	1.00			
Concurrency	0.46	0.43	-0.34	0.72	1.00		
Multiple Partners	0.66	0.38	0.00	0.76	0.84	1.00	
**South Africa**							
HIV	1.00						
HSV-2	0.96	1.00					
Syphilis	0.78	0.85	1.00				
Urethral Discharge	0.98	0.99	0.90	1.00		
Concurrency	0.86	0.92	0.99	0.95	1.00		
Multiple Partners	0.99	0.99	0.87	0.99	0.93	1.00	
**Section B**							
	HIV	HSV-2	Syphilis	Chlamydia	Gonorrhoea	Concurrency	Multiple Partners
**UK**							
HIV	1.00						
HSV-2	NA	NA					
Syphilis	0.71	NA	1.00				
Chlamydia	0.94	NA	0.90	1.00			
Gonorrhoea	0.86	NA	0.97	0.98	1.00		
Concurrency	0.91	NA	0.37	0.73	0.58	1.00	
Multiple Partners	0.96	NA	0.63	0.89	0.78	0.89	1.00
**USA**							
HIV	1.00						
HSV-2	0.99	1.00					
Syphilis	0.99	0.92	1.00				
Chlamydia	0.99	0.97	0.99	1.00			
Gonorrhoea	0.99	0.99	0.97	0.99	1.00		
Concurrency	0.89	0.68	0.91	0.84	0.79	1.00	
Multiple Partners	0.98	0.89	0.99	0.98	0.95	0.94	1.00

**Table 7.** Prevalence/incidence of HIV, syphilis, HSV-2, chlamydia, gonorrhoea, urethral discharge, genital ulcers, concurrent partners and lifetime number of partners by ethnic group in four countries and by sexual orientation in two countries. (The precise definition of each variable is provided in the methods section.).

*Country*	*Ethnic group/Sexual orientation*	*Race*	*HIV Prevalence (%)*	*Syphilis Prevalence (%)*	*HSV-2 Prevalence (%)*	*Concurrency Prevalence (%)*	*Multiple Partners (%)*	*Urethral Discharge (%)*	*Genital Ulcer (%)*	*Chlamydia Incidence*	*Gonorrhoea Incidence *	*Syphilis Incidence/Prevalence*	*Sex Partners (N)*
USA	Ethnic grp	Non-Hispanic white	0	1	12	3	15	6	NA	211	66	5	NA
USA	Ethnic grp	Hispanic	1		10	9	20	9	NA	404	114	12	NA
USA	Ethnic grp	Non-Hispanic black	2	3	39	11	27	32	NA	1176	548	24	NA
SA	Ethnic grp	White	1	0	20	2	5	2	NA	NA	NA	0	NA
SA	Ethnic grp	Coloured	3	6	30	7	8	6	NA	NA	NA	6	NA
SA	Ethnic grp	Black	20	8	61	9	16	13	NA	NA	NA	8	NA
Kenya	Ethnic grp	North East	1	1	16	NA	NA	0	NA	NA	NA	1	NA
Kenya	Ethnic grp	Central	4	1	44	15	24	1	2	NA	NA	1	NA
Kenya	Ethnic grp	Eastern	5	2	40	6	12	0	2	NA	NA	2	NA
Kenya	Ethnic grp	Western	5	1	60	19	21	2	6	NA	NA	1	NA
Kenya	Ethnic grp	Rift Valley	6	2	53	10	14	1	2	NA	NA	2	NA
Kenya	Ethnic grp	Coast	8	2	62	12	20	0	2	NA	NA	2	NA
Kenya	Ethnic grp	Nairobi	9	1	35	16	26	1	1	NA	NA	1	NA
Kenya	Ethnic grp	Nyanza	15	3	76	18	31	3	2	NA	NA	3	NA
UK	Ethnic grp	White	0	2	NA	14	30	11	NA	227	121	22	NA
UK	Ethnic grp	Asian	0	3	NA	16	23	3	NA	136	109	18	NA
UK	Ethnic grp	Black African	3	4	NA	35	44	16	NA	1011	410	29	NA
UK	Ethnic grp	Black Caribbean	3	19	NA	25	42	20	NA	1540	840	71	NA
USA	Sexual orientation	Heterosexual men	0	NA	NA	10	10	NA	NA	NA	NA	7	10
USA	Sexual orientation	MSM	7	NA	NA	31	67	NA	NA	NA	NA	233	67
UK	Sexual orientation	Heterosexual men	0	NA	NA	16	4	NA	NA	2	1	0	14
UK	Sexual orientation	MSM	6.3	NA	NA	52	24	NA	NA	24	41	7.4	111.1

***United Kingdom***

**Ethnic group** HIV prevalence varied between 2.8% in the Black Caribbean and Black African groups and 0% in the White and Asian groups ( Table 4, Figure 2). The incidence of chlamydia varied 11-fold between the Black Caribbean (1540/100 000/year) and Asian (136/100 000/year) groups. Gonorrhoea incidence varied 8-fold between these same groups (840 and 109/100 000/year, respectively). Likewise, the incidence of syphilis varied 4-fold between 71 and 18 per 100 000 per year in these same two groups. The prevalence/incidence of chlamydia, gonorrhoea, HIV and syphilis by ethnic group were positively correlated with each other (r-0.71 to r-0.98; Table 6).

The prevalence of concurrency and multiple partnering were approximately 1.5 to 2.5-fold higher in the Black African and Black Caribbean groups than the Asian and White groups. The prevalence of each STI was positively correlated with both the multiple partnering (r-0.63 to r-0.96) and concurrency (r-0.37 to r-0.91) variables.

**MSM vs heterosexuals** The prevalence of HIV was 39-fold higher in MSM outside of London (7.2%) than heterosexual men (0.16%; Table 5, Figure 3). Likewise, the incidence of chlamydia was 12-fold higher (24 vs. 2/100 000/year), gonorrhoea was 68-fold higher (41 vs. 0.6/100 000/year) and syphilis 185-fold higher (74. vs. 0.04/100 000/year) in MSM outside of London than MSW. The incidence of each of these STIs was higher in MSM residing in London. The percentage reporting concurrency was 14 times higher in MSM than heterosexuals (52% vs. 3.8%). Likewise, the mean number of lifetime partners was 8-times higher (111.1 vs. 14.3) in MSM.

### Longitudinal analyses

***UK and USA.*** In both the UK and the USA, the incidence of syphilis increased in the 1960s and 1970s but this increase was largely limited to MSM ( Figure 1) ^1, 7, 49^. The incidence remained fairly stable in heterosexuals. Although quantitative data is very limited from this period, a number of analyses attributed this increased incidence in MSM to behavioural factors, such as increases in rates of partner change ^7, 49, 55, 56^.

In the early 1980s, incidence plummeted in MSM in both countries ^1, 7^. The AIDS epidemic played a large role in this decline. Individuals most centrally placed in sexual networks were more likely to die from AIDs, which resulted in a fragmentation of sexual networks ^7, 57^. So too reductions in multiple partnering and increases in condom use both served to reduce effective network connectivity ^49, 58^. From around 2000, the incidence of syphilis has been increasing in both countries and this increase has been largely limited to MSM ^1, 7^. A very similar trend has been evident for MSM in the UK for chlamydia and gonorrhoea where reasonable surveillance data is available and is likely the case in the USA where surveillance data is less detailed as regards sexual orientation ^1, 44^. In both countries, numerous lines of evidence suggest that the introduction of effective antiretroviral therapy from 1996 onwards together with other factors resulted in increases in sexual network connectivity, which was in turn responsible for these increases in STI incidence ^1, 48^. Reduced deaths from AIDS, increases in rates of partner change and reductions in condom usage all played a role in this reconstitution of network connectivity ^7, 48, 58, 59– 61^.

**Figure 4.**
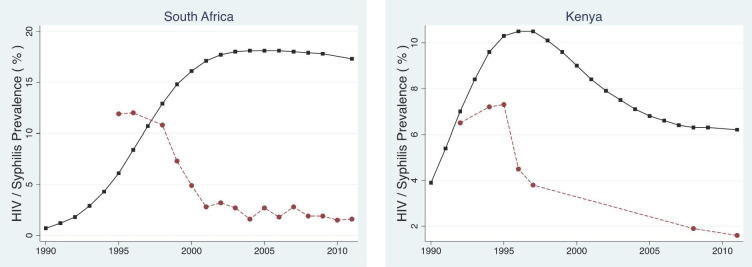
Prevalence of antenatal syphilis (red circles) and HIV (black squares) in South Africa and Kenya the United States between 1990 and 2012.

***Kenya and South Africa.*** The prevalence of antenatal syphilis in both Kenya and South Africa was between 5 and 12% in the pre-HIV period ( Figure 4; Table 3 and Table 4). A number of analyses attributed this high prevalence of syphilis to a number of factors that included behaviours that translate into dense sexual networks ^45, 62, 63^. Syphilis prevalence declined rapidly following the AIDS epidemic to below 1% in both countries ^62^. Similar, contemporaneous declines in diagnoses of cases of syphilis took place in these countries ^45, 64^. Using a range of data sources and study methodologies, a number of analyses have concluded that a combination of connectivity-reducing factors was responsible: reductions in multiple partnering (number of partners and concurrency), delayed sexual debut, increased condom usage and the effect of AIDS mortality removing more centrally placed transmission nodes ^45, 62, 65– 67^.

## Discussion

Our analysis confirmed previous analyses of dramatic variations in STI prevalence between populations ^3– 6^. A strong tendency towards clustering of STIs within subpopulations was evident. Furthermore, the cross-sectional variations in STI prevalence were almost all positively associated with two markers of network connectivity. In a similar vein, dramatic increases and declines in syphilis incidence could also be explained by corresponding changes in network connectivity. Taken together these findings suggest that network connectivity could represent a parsimonious explanation for both variations in STI incidence between populations and over time.

There are however a number of caveats to bear in mind. Our study evaluated only 4 out of over 200 countries, and the data it used was limited in a number of ways. Not all the data was from nationally representative samples. The data also came from different and limited time periods. The way the behavioural variables were defined varied between studies and in the case of Kenya, region was used as a proxy for ethnic group. For the sake of simplicity, we focused on male sexual behaviours. On the one hand, our analysis is purely ecological and therefore susceptible to the ecological inference fallacy. On the other hand, the prevalence of STIs is to a large extent a function of network level properties. Networks are inherently ecological level entities and thus ecological analyses are appropriate and necessary forms of analysis. We assume that there is a degree of segregation of sexual network by ethnic group or sexual orientation. A high degree of sexual partner homophily by ethnic group has indeed been shown for Kenya, South Africa and the USA ^4, 5, 28, 69, 70^. We could find no analyses investigating this type of homophily in the UK. If there was little or none of this type of homophily, this would however result in a homogenization of STI incidence between ethnic groups and would therefore be expected to reduce the strength of the association between STIs and behaviours by ethnic group.

A further crucial weakness is that we did not control for potential confounders. The prevalence of circumcision and condom use are two examples of factors that could confound our analyses. Other papers have, however, evaluated this question and concluded that these are likely to be only one of many determinants of differential STI prevalence ^3, 4, 31, 65^. For example, in South Africa, the highest STI prevalence ethnic group has been noted to have considerably higher circumcision rates and use of condoms than the two lower STI prevalence populations ^4, 65^. Likewise, rates of condom usage are typically higher in MSM than heterosexuals ^50^ and as far as we can ascertain, the prevalence of circumcision does not differ significantly between these two populations. A further limitation is that we have not controlled for other differences between MSM and heterosexual sexual networks such as the impact of role-versatility in MSM ^71^.

These limitations mean that we cannot conclude how generalizable our findings are or even if the associations we found are causal. What we can conclude is that our results are compatible with the network connectivity explanation. We cannot exclude the possibility that other unmeasured variables are the predominant cause.

Our findings are however logical if we recall that the number of sexual contacts per unit time is a key determinant of the rate of spread of an STI ^2^. So too increases in concurrency have been shown to lead to non-linear increases in sexual network connectivity ^9, 72^. A number of mathematical modelling studies from the USA and South Africa have established that the actual self-reported differences in number of partners and concurrency between ethnic groups in the USA and South Africa were able to explain a large part of the differential spread of HIV in these populations ^3, 65^.

A strength of our analysis is its finding that the association between network connectivity and STI prevalence held in four different countries for both comparisons by ethnic group and MSM versus heterosexuals. In the case of Kenya, all the ethnic groups/regions, including those with very low STI rates, were ‘black’ Africans. The association was found for five different STIs and in the case of syphilis, was able to explain longitudinal changes in incidence. These findings do not fit with racial theories that biological differences between races explain differential STI spread ^73^. They fit better with sociological explanations that differences in sexual behaviours result in differences in network connectivity and consequent STI incidence ^9, 74^. Furthermore, they suggest that behaviour change is possible and can result in rapid reductions in STI incidence. In fact, if network connectivity is a key determinant of STI incidence then it follows that reducing network connectivity would be an optimal way to effect radical STI prevention. Whilst high STI prevalence populations would first need to decide if they wanted to change behaviours that enhance connectivity, there are a number of positive precedents for this. Uganda’s ‘Zero grazing’ campaign was credited with large scaled reductions in multiple partnering and a subsequent decline in HIV prevalence ^75^. Similar campaigns amongst MSM in the 1980s had a similar effect ^7, 57^.

These insights are relevant in the current setting of increasing incidence of numerous STIs in high income countries ^1^. A number of analyses have found that a large proportion of these increases are occurring in MSM with high rates of partner change and particularly in contemporary HIV pre exposure prophylaxis (PrEP) recipients ^1, 38, 76^. It is not unusual for PrEP recipients to report a median of 12 partners (IQR 6–25) per 3 months ^77^. Unsurprisingly these translate into very high STI incidence - the combined incidence of gonorrhoea, chlamydia and syphilis can be up to 169 per 100 person years ^78^. Tackling this high incidence via intensive screening and treating without addressing the underlying cause may result in antimicrobial resistance ^79^.

Further studies are required to further elucidate the links between norms, structural factors, behaviour, sexual networks and STI incidence. In the interim, the type of evidence reviewed here suggests that reducing STI incidence/prevalence in high prevalence populations to those of low prevalence population may be difficult without reducing network connectivity.

## Data availability

### Underlying data

All data underlying the results are available as part of the article and no additional source data are required (see Table 1– Table 3 and Table 7).
