# Supplementary material for: Variations in sexual network connectivity may explain dramatic variations in sexually transmitted infection prevalence between populations and over time: a selected four-country analysis
Source: F1000Res. 2022 Aug 30;9:1009. Originally published 2020 Aug 20. [Version 2] doi: 10.12688/f1000research.24968.2 (PMC9490289; doi:10.12688/f1000research.24968.2)
Supplement: Peer review history

# Peer review: Reviewer response for version 2

**DOI:** 10.5256/f1000research.137876.r148907

**Published:** 2022-09-20

**Authors:** Itzchak Levy

**Affiliations:** 1 Tel-Aviv University, Tel-Aviv, Israel; 2 Sheba Medical Centre, Tel-Aviv, Israel

I would like to thank the authors for their response. There are no further comments from me.

Is the work clearly and accurately presented and does it cite the current literature?

Partly

If applicable, is the statistical analysis and its interpretation appropriate?

I cannot comment. A qualified statistician is required.

Are all the source data underlying the results available to ensure full reproducibility?

Partly

Is the study design appropriate and is the work technically sound?

Partly

Are the conclusions drawn adequately supported by the results?

Partly

Are sufficient details of methods and analysis provided to allow replication by others?

No

Reviewer Expertise:

Infectious Diseases

I confirm that I have read this submission and believe that I have an appropriate level of expertise to confirm that it is of an acceptable scientific standard.

# Peer review: Reviewer response for version 2

**DOI:** 10.5256/f1000research.137876.r148908

**Published:** 2022-09-08

**Authors:** Christine Khosropour

**Affiliations:** 1 Department of Epidemiology, University of Washington, Seattle, WA, USA

The author appropriately addressed my previous comments. I have no further comments

Is the work clearly and accurately presented and does it cite the current literature?

Yes

If applicable, is the statistical analysis and its interpretation appropriate?

I cannot comment. A qualified statistician is required.

Are all the source data underlying the results available to ensure full reproducibility?

Yes

Is the study design appropriate and is the work technically sound?

Partly

Are the conclusions drawn adequately supported by the results?

Partly

Are sufficient details of methods and analysis provided to allow replication by others?

No

Reviewer Expertise:

STI/HIV epidemiology

I confirm that I have read this submission and believe that I have an appropriate level of expertise to confirm that it is of an acceptable scientific standard.

# Peer review: Reviewer response for version 1

**DOI:** 10.5256/f1000research.27545.r142231

**Published:** 2022-07-18

**Authors:** Itzchak Levy

**Affiliations:** 1 Tel-Aviv University, Tel-Aviv, Israel; 2 Sheba Medical Centre, Tel-Aviv, Israel

The study done by Chris Kenyon is very interesting and important. It tries to show the association between the connectivity within a group (which in this work is expressed by the number of partners in a certain time and the concurrency of partners) and the incidence/prevalence of certain sexually transmitted infections (STIs). This is important because understanding sexual networks and how they work and what influences them is very important in the prevention of STIs. It adds to the knowledge we get from the study of social and sexual networks as well to phylogenetic studies of the spread of STIs.

 However, since this paper is constructed from many different works that studied different questions (incidence of some STIs, prevalence of STIs, sexual behavior data, etc.) as a clinician I don't understand how the conclusions of the writer can be made and I feel that the input of a statistician would be much more helpful.

 It sounds more reasonable to take one or two STIs which are compatible with a dynamic study (e.g. gonorrhea and chlamydia) and to concentrate on a more defined population. However, this study still gives the "wide picture".

 HIV in my opinion is not suitable for this study because its prevalence is influenced not only by the incidence but also by some other factors such as the introduction of anti-retroviral therapy (ART) in 1996 and the introduction of better treatments like integrase inhibitors in the 2000s. Incidence may be also influenced by introduction of earlier treatment (treatment as prevention) and introduction of pre exposure prophylaxis (PREP) so it is also less suitable for this study.

 The methods section should be somewhat explained better. It is not understandable if the work is a systematic review of the literature, then it would be helpful if you can explain the method by which the specific studies were chosen and why others were not (a flow chat may be helpful).

 There is a mixture of studies, some show incidence and some show prevalence – this mixture is confusing.

 With such a big population I don't quite understand what is the meaning of the correlation.

 There are a lot of prevalence studies which utilize serology like studies 42 and 43 (HSV2 and syphilis). There is no way to know the date of infection in those studies so it is a very big limitation to use them for the purpose of this study which tries to show dynamics.

 In Kenya (page 6) ethnicity was a region of residence served as a proxy for ethnicity. I am not sure that this approximation can be done safely for the purpose of this study because it is well established that MSM tend to reside in big cities without regard to their ethnicity so I would leave it as "regional".

 In Figure 2 (page 7) The rate of MSM in each ethnic group is not given and this may influence the data and the conclusions.

 In Figure 4 legend (page 9) the word the united states should be omitted.

Is the work clearly and accurately presented and does it cite the current literature?

Partly

If applicable, is the statistical analysis and its interpretation appropriate?

I cannot comment. A qualified statistician is required.

Are all the source data underlying the results available to ensure full reproducibility?

Partly

Is the study design appropriate and is the work technically sound?

Partly

Are the conclusions drawn adequately supported by the results?

Partly

Are sufficient details of methods and analysis provided to allow replication by others?

No

Reviewer Expertise:

Infectious Diseases

I confirm that I have read this submission and believe that I have an appropriate level of expertise to confirm that it is of an acceptable scientific standard, however I have significant reservations, as outlined above.

# Response

**Published:** 2022-08-24

**Authors:** Chris Kenyon

**Affiliations:** Institute of Tropical Medicine, Antwerp, Belgium

Reply to reviewer 2:

 The study done by Chris Kenyon is very interesting and important. It tries to show the association between the connectivity within a group (which in this work is expressed by the number of partners in a certain time and the concurrency of partners) and the incidence/prevalence of certain sexually transmitted infections (STIs). This is important because understanding sexual networks and how they work and what influences them is very important in the prevention of STIs. It adds to the knowledge we get from the study of social and sexual networks as well to phylogenetic studies of the spread of STIs.

 However, since this paper is constructed from many different works that studied different questions (incidence of some STIs, prevalence of STIs, sexual behavior data, etc.) as a clinician I don't understand how the conclusions of the writer can be made and I feel that the input of a statistician would be much more helpful.

 It sounds more reasonable to take one or two STIs which are compatible with a dynamic study (e.g. gonorrhea and chlamydia) and to concentrate on a more defined population. However, this study still gives the "wide picture".

 HIV in my opinion is not suitable for this study because its prevalence is influenced not only by the incidence but also by some other factors such as the introduction of anti-retroviral therapy (ART) in 1996 and the introduction of better treatments like integrase inhibitors in the 2000s. Incidence may be also influenced by introduction of earlier treatment (treatment as prevention) and introduction of pre exposure prophylaxis (PREP) so it is also less suitable for this study.

**Reply:**

**Thank you for these useful comments. The limitations section has been expanded to include the concerns raised by the reviewer (P 23, second paragraph):**

*Differential uptake of antiretroviral therapy is an important potential determinant of differential HIV prevalence between sub-populations. Once again, we did not control for this.*

*It should be noted that in the case of South Africa the differential HIV prevalence between ethnic groups was of a similar magnitude in surveys done before ART became available to those following the widespread use of ART.*

 The methods section should be somewhat explained better. It is not understandable if the work is a systematic review of the literature, then it would be helpful if you can explain the method by which the specific studies were chosen and why others were not (a flow chat may be helpful).

**Reply:**

**As noted above the countries were not selected via a systematic review methodology. The rationale for selecting the studies has been added to the introduction.  This limitation has been added to the relevant section of the limitations in the discussion (P 23, 3 ^rd^ Paragraph).**

**In addition, the following sentence has been added to the introduction to make it clear how the four countries were chosen (P3, third last sentence).**

**These countries were chosen due to the familiarity of the author with these countries and the availability of nationally representative datasets with both sexual behaviour and STI prevalence data.**

**Finally, we have changed the title of the paper from “a four-country analysis” to “a selected four-country analysis.”**

 There is a mixture of studies, some show incidence and some show prevalence – this mixture is confusing.

 With such a big population I don't quite understand what is the meaning of the correlation.

 There are a lot of prevalence studies which utilize serology like studies 42 and 43 (HSV2 and syphilis). There is no way to know the date of infection in those studies so it is a very big limitation to use them for the purpose of this study which tries to show dynamics.

**Reply:**

**It is true that one cannot know the date of infection in studies that define STI incidence/prevalence with HIV, HSV-2 serology or a treponemal test only in the case of syphilis. These variables are then best considered as a measure of cumulative incidence. A number of studies have found that these variables offer a valuable marker of how connected sexual networks are and what their risk is for other STIs** **(1, 2)** **. As an example, the prevalence of HSV-2 in 20-24 year olds can predict subsequent HIV prevalence** **(2)** **. The same might be true for emergent STIs such as monkeypox. For most of the studies of syphilis incidence/prevalence used in this paper, syphilis was defined by both a treponemal and non-treponemal test.**

 In Kenya (page 6) ethnicity was a region of residence served as a proxy for ethnicity. I am not sure that this approximation can be done safely for the purpose of this study because it is well established that MSM tend to reside in big cities without regard to their ethnicity so I would leave it as "regional".

 In Figure 2 (page 7) The rate of MSM in each ethnic group is not given and this may influence the data and the conclusions.

**Reply:**

**We provide a justification for the use of regions as a proxy for ethnicity in Kenya in the Methods section (P 11, third paragraph):**

*In addition, we have added this as a limitation in the discussion section (P 23, second paragraph):*

*The way the behavioural variables were defined varied between studies and in the case of Kenya, region was used as a proxy for ethnic group*

***Regions vs. ethnic group in Kenya. **Many of the national STI surveillance surveys in Kenya do not collect data on ethnic group ^29 ^. There is however a high correlation between region of residence and ethnic group, with large differences in STI incidence and sexual behaviour between regions ^5, 31 ^. We therefore follow the approach used in previous studies and use region of residence as a proxy for ethnic group ^31, 54 ^.*

 In Figure 4 legend (page 9) the word the united states should be omitted.

**Reply:**

**Thanks for pointing out this error, which has been corrected.**

 References

 1.         Kenyon C. Strong associations between national prevalence of various STIs suggests sexual network connectivity is a common underpinning risk factor. BMC Infect Dis. 2017;17(1):682.

 2.         Kenyon C, Colebunders R, Hens N. Determinants of generalized herpes simplex virus-2 epidemics: the role of sexual partner concurrency. Int J STD AIDS. 2013;24(5):375-82.

# Peer review: Reviewer response for version 1

**DOI:** 10.5256/f1000research.27545.r92342

**Published:** 2021-09-08

**Authors:** Christine Khosropour

**Affiliations:** 1 Department of Epidemiology, University of Washington, Seattle, WA, USA

In this review, Dr. Kenyon reviews data on STI/HIV incidence/prevalence and partner concurrency from (mostly) population-based studies in four counties. The results show that STIs cluster within certain groups defined by race/ethnicity and gender of sex partners, and that STIs were correlated with number of sex partners and concurrency. It is helpful to have these data summarized for these countries. The author appropriately addresses the limitations. My main comment has to do with the transparency in how these papers were selected. I think adding in the full search criteria, the full exclusion/inclusion criteria, the number of studies identified and eliminated, etc would be appropriate here. I also had trouble identifying which data supported which conclusions. These and other comments are detailed below.

**Minor comments:**

1. Could the author specify upfront their use of sex/gender terms throughout the paper? Even just to say that the terms they use reflect those from published papers, where it may have been unclear whether the studies referred to sex at birth or gender identity.
2. The author presents data stratified by “ethnic groups”. Could the author provide some description in the methods about how stratification of these groups was operationalized, by country? There is some of that for Kenya already.
3. Page 5 of the PDF, second to last paragraph: The author states: “these variations in definitions between studies mean it would be appropriate to compare these variables across studies”. Did the author mean to say “it would be *inappropriate*”?
4. Page 8 of the PDF, first paragraph: I believe the reference should be to Figure 2, not Figure 1.
5. The author uses the terms “heterosexual men” and “men who have sex with women” (Table 2). I think it would be helpful to choose one throughout. There are also places where the author states “heterosexuals” which I assume to be men only but it is a little unclear
6. There appears to be a typo in the Figure 4 title (the phrase “the United States” should not be there)

**Major comments:**

1. The paper is not described as a systematic review, but was it intended to be? Given that there are summary quantitative estimates provided, it feels like a systematic review. If so, I think it would have been helpful in the results to see how many papers were identified using the search criteria and how many were eliminated, and for what reasons. When I ran this search (using “or” between the terms but also making sure the countries were included) I get >225,000 results. I assume the author used a different order of terms. Could the full order of search terms be included in the paper?
2. Somewhat related to the above, I am having a bit of trouble figuring out how studies were selected. For example, it appears that there were only 3 studies from Kenya published since 1990 that make comparisons on the prevalence of STIs/HIV, multiple partners, or concurrency. Is that the case? Or were these the only population-based studies? I think adding in inclusion/exclusion criteria would be helpful.
3. To calculate chi-square values for incidence/prevalence, did the author pool raw numbers from different studies or use some other method? Would there be potential for violation of an assumption of independence of observations?
4. Figure 3: I do not understand the primary Y axis here (labeled “HIV, syphilis, concurrency, lifetime partners). Is this the percentage of individuals who reported living with HIV, being diagnosed with syphilis, reporting concurrency, or reporting over a certain number of partners?
5. The discussion focuses on the relationship between STIs and concurrency. Unless I misunderstand the data, it seems that these data are mostly from Table 6, which shows the correlation of STIs and concurrency and multiple partners. To me, it does not look like the associations are particularly strong in Kenya, and it does not really hold for syphilis in the UK or gonorrhea in the UK. Are there other data from which the author is drawing to make these conclusions?
6. Page 14 of the PDF, first paragraph: I would change the phrase: “These findings do not fit with racial theories that biological differences...” to ““These findings do not fit with *racist* theories that biological differences...”
7. Page 14 of the PDF, first paragraph: The author suggests reducing network connectivity as a way to decrease STI spread. I do not understand how that would be operationalized. Is the author referring to reducing concurrency? I am not familiar with the Zero grazing campaign; perhaps that could be explained more to help the reader understand the concept of “breaking up” network connectivity
8. In the conclusion, I think it may be worth adding a comment about investment in STI vaccines being a plausible solution to curbing STI rates. Probably a more viable solution than behavior change.

Is the work clearly and accurately presented and does it cite the current literature?

Yes

If applicable, is the statistical analysis and its interpretation appropriate?

I cannot comment. A qualified statistician is required.

Are all the source data underlying the results available to ensure full reproducibility?

Yes

Is the study design appropriate and is the work technically sound?

Partly

Are the conclusions drawn adequately supported by the results?

Partly

Are sufficient details of methods and analysis provided to allow replication by others?

No

Reviewer Expertise:

STI/HIV epidemiology

I confirm that I have read this submission and believe that I have an appropriate level of expertise to confirm that it is of an acceptable scientific standard, however I have significant reservations, as outlined above.

# Response

**Published:** 2022-08-24

**Authors:** Chris Kenyon

**Affiliations:** Institute of Tropical Medicine, Antwerp, Belgium

Reply to reviewer 1:

 1 In this review, Dr. Kenyon reviews data on STI/HIV incidence/prevalence and partner concurrency from (mostly) population-based studies in four counties. The results show that STIs cluster within certain groups defined by race/ethnicity and gender of sex partners, and that STIs were correlated with number of sex partners and concurrency. It is helpful to have these data summarized for these countries. The author appropriately addresses the limitations. My main comment has to do with the transparency in how these papers were selected. I think adding in the full search criteria, the full exclusion/inclusion criteria, the number of studies identified and eliminated, etc would be appropriate here. I also had trouble identifying which data supported which conclusions. These and other comments are detailed below.

**Reply:**

**The following sentence has been added to the introduction to make it clear how the four countries were chosen (P3, third last sentence).**

**These countries were chosen due to the familiarity of the author with these countries and the availability of nationally representative datasets with both sexual behaviour and STI prevalence data.**

**Minor comments:**

- Could the author specify upfront their use of sex/gender terms throughout the paper? Even just to say that the terms they use reflect those from published papers, where it may have been unclear whether the studies referred to sex at birth or gender identity.

**Reply:   A new section “Terminology” has been added to the Methods section to address this point:**

**Sex, gender, ethnicity and race: Where this was specified, each data source used a slightly different definition of sex, gender, ethnicity and race. Wherever possible, we used the same terms used in these original sources.**

  

- The author presents data stratified by “ethnic groups”. Could the author provide some description in the methods about how stratification of these groups was operationalized, by country? There is some of that for Kenya already.

**Reply: A new section to the methods section has been added to address this question (P11, penultimate paragraph).**

- Page 5 of the PDF, second to last paragraph: The author states: “these variations in definitions between studies mean it would be appropriate to compare these variables across studies”. Did the author mean to say “it would be *inappropriate*”

**Reply: Thanks for pointing this typo out. It has been corrected. **

- Page 8 of the PDF, first paragraph: I believe the reference should be to Figure 2, not Figure 1.

**Reply: Thanks for pointing this error out. It has been corrected. **

- The author uses the terms “heterosexual men” and “men who have sex with women” (Table 2). I think it would be helpful to choose one throughout. There are also places where the author states “heterosexuals” which I assume to be men only but it is a little unclear

**Reply: The phrase “men who have sex with women” has been deleted everywhere and replaced with “heterosexual men.”**

- There appears to be a typo in the Figure 4 title (the phrase “the United States” should not be there)

**Reply: Thanks for pointing this error out. It has been corrected.**

**Major comments:**

- The paper is not described as a systematic review, but was it intended to be? Given that there are summary quantitative estimates provided, it feels like a systematic review. If so, I think it would have been helpful in the results to see how many papers were identified using the search criteria and how many were eliminated, and for what reasons. When I ran this search (using “or” between the terms but also making sure the countries were included) I get >225,000 results. I assume the author used a different order of terms. Could the full order of search terms be included in the paper?

** Reply: As noted above the countries were not selected via a systematic review methodology. The rationale for selecting the studies has been added to the introduction.  This limitation has been added to the relevant section of the discussion (P 23, 3 ^rd^ Paragraph).**

- Somewhat related to the above, I am having a bit of trouble figuring out how studies were selected. For example, it appears that there were only 3 studies from Kenya published since 1990 that make comparisons on the prevalence of STIs/HIV, multiple partners, or concurrency. Is that the case? Or were these the only population-based studies? I think adding in inclusion/exclusion criteria would be helpful.

**Reply:**

**Where possible surveys using nationally representative samples were used. Where this data was not available other sources were used such as detailed in Table 2. This information has been added to the methods section. **

- To calculate chi-square values for incidence/prevalence, did the author pool raw numbers from different studies or use some other method? Would there be potential for violation of an assumption of independence of observations?

**Reply: Chi-squared tests were performed in the raw numbers per group. It is acknowledged that this approach is of limited utility and if the reviewer prefers, these statistical tests could be removed.**

- Figure 3: I do not understand the primary Y axis here (labeled “HIV, syphilis, concurrency, lifetime partners). Is this the percentage of individuals who reported living with HIV, being diagnosed with syphilis, reporting concurrency, or reporting over a certain number of partners?

**Reply: These are defined in the Figure 3 legend as follows: HIV and partner concurrency prevalence are reported as percentages, multiple partners as the number of lifetime partners and chlamydia, gonorrhoea and syphilis incidence as cases per 100 000 per year.**

  

- The discussion focuses on the relationship between STIs and concurrency. Unless I misunderstand the data, it seems that these data are mostly from Table 6, which shows the correlation of STIs and concurrency and multiple partners. To me, it does not look like the associations are particularly strong in Kenya, and it does not really hold for syphilis in the UK or gonorrhea in the UK. Are there other data from which the author is drawing to make these conclusions?

**Reply: The data referred to is that from Table 6. I agree with the reviewer’s comments and have added this concern to the list of limitations (P23, third paragraph):**

**Not all the associations between multiple partnering/ concurrency and STI incidence/prevalence were strongly positive. In fact, one association (between syphilis and concurrency by ethnic group in Kenya) was weakly negative (Table 6).**

- Page 14 of the PDF, first paragraph: I would change the phrase: “These findings do not fit with racial theories that biological differences...” to ““These findings do not fit with *racist* theories that biological differences...”

**Reply: This change has been made. **

- Page 14 of the PDF, first paragraph: The author suggests reducing network connectivity as a way to decrease STI spread. I do not understand how that would be operationalized. Is the author referring to reducing concurrency? I am not familiar with the Zero grazing campaign; perhaps that could be explained more to help the reader understand the concept of “breaking up” network connectivity

**Reply: This section has been expanded as suggested.**

  

- In the conclusion, I think it may be worth adding a comment about investment in STI vaccines being a plausible solution to curbing STI rates. Probably a more viable solution than behavior change.

**Reply: Thank you for this point, which has been added to the last sentence of the paper.**
